# *Furfurilactobacillus* *entadae* sp. nov., Isolated from Bark of *Entada phaseoloides*

**DOI:** 10.1007/s00284-025-04450-4

**Published:** 2025-08-29

**Authors:** Shunya Suzuki, Karin Okano, Mizuna Tamaki, Yoshimasa Tsujii, Akihito Endo, Akinobu Kajikawa

**Affiliations:** 1 Department of Agricultural Chemistry, Graduate School of Tokyo University of Agriculture, 1-1-1 Sakuragaoka, Setagaya, Tokyo 156-8502 Japan; 2 Bioproduction Research Institute, National Institute of Advanced Industrial Science and Technology, 1-1-1 Higashi, Tsukuba, Ibaraki 305-8566 Japan; 3 Department of Nutritional Science and Food Safety, Faculty of Applied Bioscience, Tokyo University of Agriculture, 1-1-1 Sakuragaoka, Setagaya, Tokyo 156-8502 Japan

## Abstract

A novel lactic acid bacterium, OKN36^T^, was isolated from the bark of *Entada phaseoloides* on Amami Island in Japan. Strain OKN36^T^ is characterized as a Gram-stain-positive, non-motile, catalase-negative, heterofermentative, and rod-shaped bacterium. The strain grew at temperatures ranging from 10 to 37 °C, in a pH range of 4.0–8.0, and in the presence of 0–6.5% (w/v) NaCl. Phylogenetic analysis using the 16S rRNA gene analysis revealed that strain OKN36^T^ belongs to the *Furfurilactobacillus* group. The average nucleotide identity (ANI) and the digital DNA–DNA hybridization (dDDH) values between strain OKN36^T^ and type strains of validly published *Furfurilactobacillus* species were 71.83–73.85% and 19.7–20.6%, respectively. These values are below the cutoff of species differentiation, which is 95–96% for ANI and 70% for dDDH. Based on these data, we propose a novel species, named *Furfurilactobacillus entadae* sp. nov., with the type strain OKN36^T^ (= JCM 37107^T^ = DSM 118293^T^).

**Supplementary Information:**

The online version contains supplementary material available at 10.1007/s00284-025-04450-4.

## Introduction

In 2020, the genus *Lactobacillus* was reclassified, and the former *Lactobacillus rossiae* group was newly designated as the genus *Furfurilactobacillus* [1]. Currently, the genus *Furfurilactobacillus* includes four species: *Furfurilactobacillus rossiae*, *Furfurilactobacillus siliginis*, *Furfurilactobacillus curtus*, and *Furfurilactobacillus milii*. These furfurilactobacilli were mainly isolated from fermented foods, such as *F. rossiae*, *F. siliginis*, *F. milii* from sourdough [2–4], *F. curtus* from beer [5], and *F. rossiae* from fermented meat [6]. They appear to be predominantly associated with environmental and fermented food niches [7, 8]. Some *Furfurilactobacillus* species exhibit beneficial properties for food fermentation, including antifungal activity and phenolic compound metabolism [9–12]. Despite their potential in food fermentation, the ecology of the genus *Furfurilactobacillus* remains largely unknown due to the limited number of isolated species within the genus.

In this study, one strain, designated OKN36^T^, was isolated from the bark of *Entada phaseoloides* in Japan and characterized by phenotypic and genotypic analysis. Based on the results, we propose this strain a novel *Furfurilactobacillus* species, named *Furfurilactobacillus entadae* sp. nov.,

## Material and Methods

### Isolation

Stain OKN36^T^ was isolated from the bark of *Entada phaseoloides* collected at Amami Island, Japan, in November 2020. The bark was placed in a sterile conical tube with Man–Rogosa–Sharpe (MRS) broth. After incubation at 30 °C, the suspension was serially diluted and plated onto MRS agar plates. The agar plates were incubated at 30 °C under anaerobic conditions using AnaeroPouch-Anaero Anaerobic Gas Generators (Mitsubishi Gas Chemical). After isolation by repetitive dilution streaks, the strain was cultured in MRS broth and stored at − 80 °C in MRS broth containing 20% (v/v) glycerol. Among the isolates obtained from the bark sample, strain OKN36^T^ was selected for further characterization because its 16S rRNA gene sequence showed low similarity to type strains of species with validly published name.

### 16S rRNA Gene Phylogeny

The 16S rRNA gene sequence of the isolate was determined according to a previously described method [13]. Briefly, the 16S rRNA gene sequence of strain OKN36^T^ was amplified with 16S primers, 27F and 1492R, and Ex *Taq* DNA polymerase (Takara). The PCR product was purified using the NucleoSpin Gel and PCR Clean-up kit (Macherey–Nagel) according to the manufacturer’s instructions and sequenced by Macrogen Japan (Tokyo, Japan) with the primers 27F and 1492R. The GenBank database was used for BLAST analysis of the 16S rRNA gene sequence. Multiple sequence alignment was carried out with ClustalW [14]. Distance matrices for the aligned sequences were calculated using the two-parameter method of Kimura [15]. The phylogenetic tree was generated with MEGA X [16], using the maximum-likelihood and neighbor-joining methods with 1000 bootstrap replications, and visualized using Interactive Tree of Life (iTOL) v6 [17]. *Lactiplantibacillus plantarum* WCFS1 (KC429782.1) was used as an outgroup.

### Genome Features

For genome sequencing, genomic DNA from strain OKN36^T^ and *F. curtus* JCM 31185^T^ was extracted using DNAiso Reagent (Takara) according to the manufacturer’s instructions. Whole-genome sequencing was performed using Illumina Hiseq by Eurofins Genomics (Tokyo, Japan). Draft genomes were assembled using Platanus_B [18] with default settings. Sequences shorter than 300 bp were eliminated. The genome was annotated using the DDBJ Fast Annotation and Submission Tool (DFAST, https://dfast.nig.ac.jp) [19]. The completeness and contamination of the genomic data were assessed by CheckM [20]. Average nucleotide identity (ANI) was calculated as the mean identity of pair-wise sequence alignment between two genomes [21]. Digital DNA–DNA hybridization (dDDH) values were determined by Genome-to-Genome Distance Calculator (http://ggdc.dsmz.de) [22]. For further species delimitation, a core-genome phylogenetic tree was reconstructed as described previously [23]. Orthologous clusters that were conserved in the genus *Furfurilactobacillus* and related taxa and *L. plantarum* WCFS1 (GCA000203855.3, included as the out-group) were determined by GET_ HOMOLOGUES software based on the all-against-all bidirectional BLAST alignment and the MCL graph-based algorithm [24]. The amino acid sequences within each cluster were aligned using MUSCLE [25]. Poorly aligned or divergent regions were trimmed using trimAI [26], and conserved regions were then concatenated using FASconCAT-G [27]. A partitioned maximum-likelihood analysis was performed to construct the phylogenetic tree with IQ-TREE2 [28] and visualized using iTOL v6 [17]. The number of bootstrapping was 1000 replicates.

### Physiology and Chemotaxonomy

The type strains of *F. rossiae*, *F. siliginis*, and *F. curtus* were purchased from RIKEN BioResource Center, Ibaraki, Japan. Strain OKN36^T^ and the type strains of *F. rossiae*, *F. siliginis*, and *F. curtus* were anaerobically grown in MRS broth or agar plates at 30 °C. Cells from cultures grown for 24 h were tested for Gram staining. The morphology was observed using a BZ-X710 microscope (KEYENCE, Osaka, Japan). The motility of the strains was determined by observation after growth in a semi-solid MRS medium with 0.3% (w/v) agar. Strain OKN36^T^ and the type strains of *F. rossiae*, *F. siliginis*, and *F. curtus* were individually grown in MRS broth with various conditions such as different temperatures (10, 15, 20, 25, 30, 35, 37, 40 and 45 °C), pH (pH 3.5, 4.0, 4.5, 5.0, 6.0, 7.0, 7.5, 8.0, 8.5 and 9.0), and NaCl salt concentrations (0, 3.0, 4.0, 5.0, 6.0, 6.5, 7.5, 10, 12.5, 15, 18 and 20%). Acid production from carbohydrates was determined using the API 50 CH kit (bioMérieux) according to the manufacturer’s instructions. Supplementary phenotypic and biochemical properties, such as gas production in MRS broth, catalase activity, dextran production from sucrose, and d/l-lactate formation were analyzed according to the standard protocols. Cellular fatty acids were extracted from strain OKN36^T^ and converted into fatty acid methyl esters according to the method described by Sakamoto et al. (2002) [29]. The fatty acids were analyzed using gas chromatography and identified by comparison with the NIST compound library.

## Results and Discussion

### 16S rRNA Gene Phylogeny

**Fig. 1:**
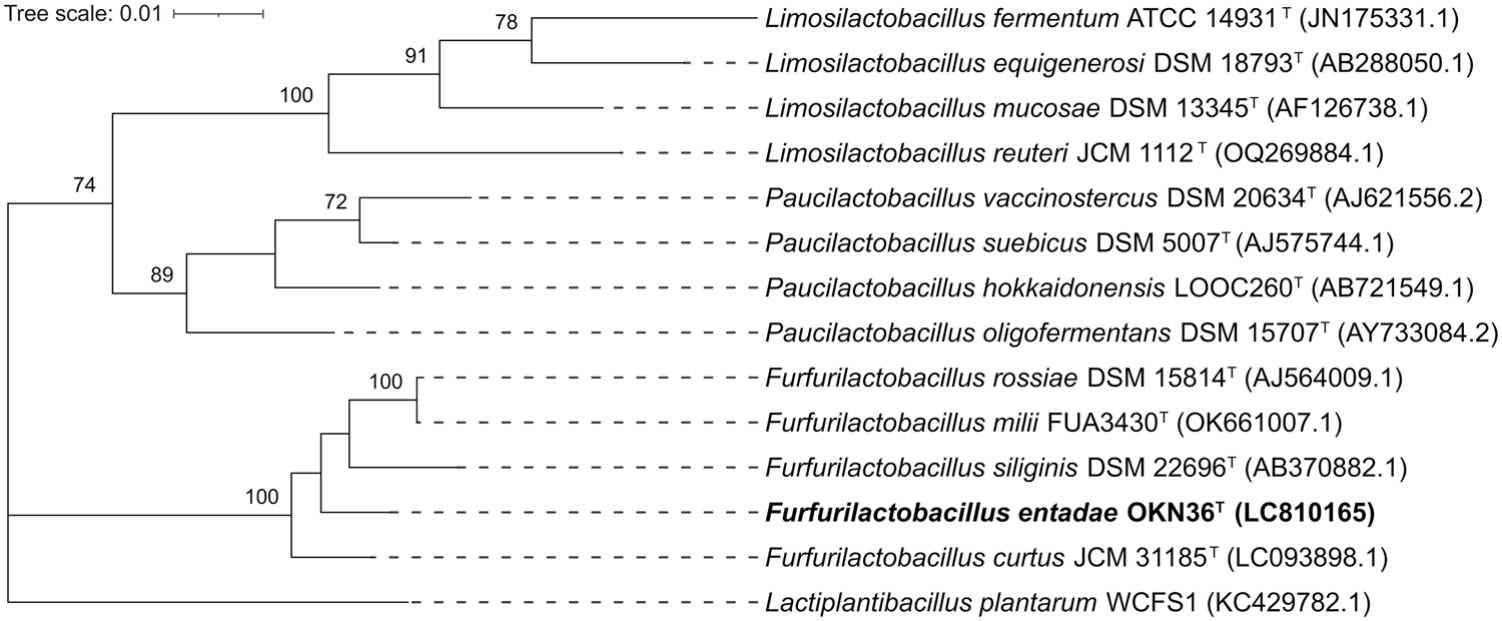
Phylogenetic tree of *Furfurilactobacillus entadae* OKN36^T^ and related taxa based on 16S rRNA gene sequences. The tree was constructed using the maximum-likelihood method. The values on the branches are bootstrap support from 1000 rapid bootstrapping replicates and only values over 70% are indicated. *Lactiplantibacillus plantarum* WCFS1 (KC429782.1) was used as an outgroup. The scale bar means substitution per site

The almost complete 16S rRNA gene sequences of the strain OKN36^T^ (1453 bp, LC810165) was determined by the Sanger sequencing. The result of the BLAST analysis revealed that the highest sequence similarities to strain OKN36^T^ were 98.15, 98.08, and 98.01% to *F. rossiae*, *F. milii*, and *F. siliginis*, respectively. In the phylogenetic trees based on 16S rRNA gene sequences reconstructed using the maximum-likelihood and neighbor-joining methods, the strain formed a cluster with the type strains of *F. rossiae*, *F. siliginis*, *F. curtus,* and *F. milii* (Figs. 1 and S1). These results indicated that strain OKN36^T^ is included in the *Furfurilactobacillus* clade.

### Genome Features

**Fig. 2:**
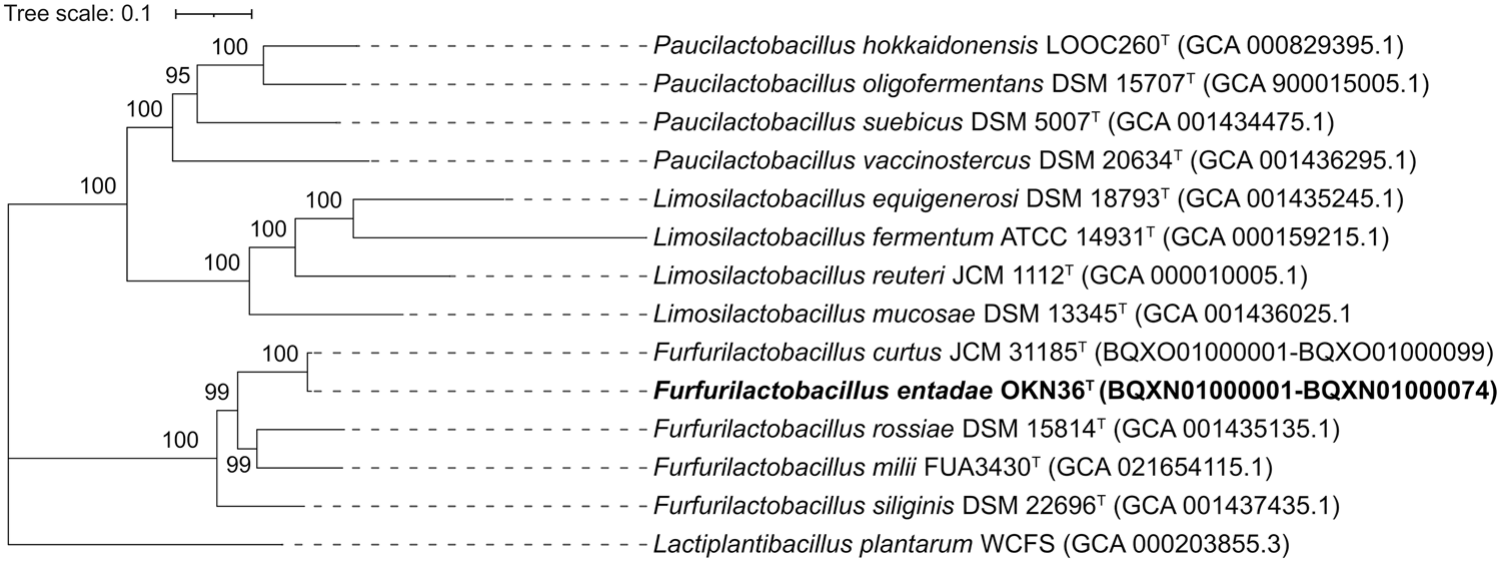
Core-genome phylogenetic tree of *Furfurilactobacillus entadae* OKN36^T^ and related taxa based on multiple alignments of protein sequences for the 594 single-copy genes. The maximum-likelihood tree was constructed using IQ-TREE2. The values on the branches are bootstrap support from 1000 rapid bootstrapping replicates, and only values over 90% are indicated. *Lactiplantibacillus plantarum* WCFS1 (GCA000203855.3) was used as an outgroup. The scale bar means substitution per site

The genome size of strain OKN36^T^ was 2.6 Mbp, containing 2246 cording sequences (Table 1). The molar G + C content was 46.8%. As summarized in Table 2, the ANI and dDDH values for the type strains of all *Furfurilactobacillus* ranged from 71.83–73.85% to 19.7–20.6%, respectively. These values were lower than the cutoff values of species demarcation, which are 95–96% (ANI) and 70% (dDDH). The core-genome tree showed that stain OKN36^T^ formed a cluster with the type strains of *F. rossiae*, *F. siliginis*, *F. curtus,* and *F. milii* (Fig. 2). In the *Furfurilactobacillus* phylogenetic cluster, strain OKN36^T^ positioned distantly to other strains. These genomic data indicate that strain OKN36^T^ represents a novel species of the genus *Furfurilactobacillus*.

**Table 1:** Genomic features of the strains used in this study

Strain	Genome size (Mbp)	No. of CDS	G + C content (%)	Genome accession
*F. rossiae* DSM 15814^T^	2.9	2641	43.3	GCA_001435135.1
*F. siliginis* DSM 22696^T^	2.1	1970	44.1	GCA_001437435.1
*F. curtus* JCM 31185^T^	2.2	1982	43.9	BQXO01000001-BQXO01000099
*F. milii* FUA3430^T^	2.6	2442	43.5	GCF_021654115.1
*F. entadae* OKN36^T^	2.3	2246	46.8	BQXN01000001-BQXN01000074

**Table 2:** ANI (top right) and dDDH (bottom left) values among *Furfurilactobacillus* strains

Strain	DSM 15814^T^	DSM 22696^T^	JCM 31185^T^	FUA3430^T^	OKN36^T^
*F. rossiae* DSM 15814^T^	–	74.12	72.64	92.63	73.21
*F. siliginis* DSM 22696^T^	20.9	–	72.71	74.09	73.85
*F. curtus* JCM 31185^T^	21.1	22.8	–	72.63	71.83
*F. milii* FUA3430^T^	49.0	22.2	22.0	–	73.02
*F. entadae* OKN36^T^	20.2	20.6	19.7	20.1	–

### Phenotypic Properties

The colonies of strain OKN36^T^ were approximately 1 mm in diameter, white, smooth, and circular. The cells of strain OKN36^T^ and the type strains of *F. rossiae*, *F. siliginis*, and *F. curtus* were Gram-stain-positive, non-motile, short round rods. As summarized in Table 3, strain OKN36^T^ was able to grow at temperatures from 10 to 37 °C with optimum growth at 35 °C. Strain OKN36^T^ grew in the pH range of 4.0–8.0, and the optimum pH was pH 6.0. The strain also grew with salt concentrations between 0 and 6.5%, with an optimal salt concentration of 0%. All the strains could produce acid from d-ribose, d-glucose, d-mannose, d-maltose, *N*-acetyl-glucosamine, aesculin, d-maltose, and gluconate, but not glycerol, erythritol, d-arabinose, l-xylose, d-adonitol, methyl *β*-d-xylopyranoside, l-sorbose, l-rhamnose, dulcitol, inositol, d-mannitol, d-sorbitol, methyl-*α*-d-mannopyranoside, methyl *α*-d-glucopyranoside, amygdalin, arbutin, salicin, d-cellobiose, d-lactose, d-sucrose, d-trehalose, inulin, d-melezitose, d-raffinose, starch, glycogen, xylitol, gentiobiose, d-turanose, d-lyxose, d-tagatose, d-fucose, l-fucose, l-arabitol, 2-ketogluconate, and 5-ketogluconate. Different patterns of acid production from l-arabinose, d-xylose, d-galactose, d-fructose, d-mannose, *N*-acetyl-glucosamine, d-melibiose, and gluconate were shown among the tested strains (Table 3). Supplementary phenotypic and biochemical properties were analyzed according to the standard protocols. The results show that strain OKN36^T^ is catalase-negative, heterofermentative, and produces both isomers of lactate. Nitrate is not reduced, and dextran is not produced from sucrose. The major cellular fatty acids are C_16:0_, C_18:1_, and C_19_ (Table 3).

**Table 3:** Differential phenotypic characteristics of *F. entadae* OKN36^T^ and related species of the genus *Furfurilactobacillus*

Characteristics	1	2	3	4	5
Isolation source	Tree	Sourdough	Sourdough	Beer	Sourdough
Motility	–	–	–	–	ND
Catalase reaction	–	–	–	–	ND
Growth temperature (°C)	10–37	15–40	10–30	10–37	15–37
Growth pH	4.0–8.0	3.5–7.5	4.5–7.5	4.0–7.5	5.0–9.0
Major fatty acids	C_16:0_, C_18:1_, C_19_	ND	ND	ND	C_19_ *cyc* 9, C_10_, C_18: 1_ *cis* 9, C_16: 1_ *cis* 9, C_16: 0_ and C_18: 1_ *cis* 10
Acid production from:					
l-Arabinose	W	+	–	–	+
d-Xylose	–	+	–	+	–
d-Galactose	W	W	W	–	+
d-Fructose	+	+	–	+	+
d-Mannose	W	W	W	+	+
*N*-Acetyl-glucosamine	W	W	+	W	+
d-Melibiose	–	–	–	+	–
d-Arabitol	–	–	–	+	–
Gluconate	W	W	W	+	+

Strains: 1, *F. entadae* OKN36^T^; 2, *F. rossiae* DSM 15814^T^; 3, *F. siliginis* DSM 22696^T^; 4, *F. curtus* JCM 31185^T^; 5, *F. milii* FUA3430^T^. Data for the type strain of *F. milii* are taken from [4]

### Taxonomic Conclusions

The data show that strain OKN36^T^ is phylogenetically and biochemically distinct from species with validly published name in the genus *Furfurilactobacillus*. Thus, the strain represents a novel species for which the name *Furfurilactobacillus entadae* sp. nov. is proposed. The type strain is OKN36^T^ (= JCM 37107^T^ = DSM 118293^T^).

### Description of Furfurilactobacillus entadae sp. nov.

*Furfurilactobacillus entadae* (en.ta’dae. N.L. gen. n. *entadae*, of *Entada*, a genus of leguminous plants from which the type strain was isolated).

Cells are facultatively anaerobic, Gram-positive, catalase-negative, and non-motile rods. Colonies anaerobically incubated on MRS agar (2–3 days at 30 °C) are approximately 1 mm in diameter, white, smooth, and circular. Gas is produced from d-glucose, thus being heterofermentative. Nitrate is not reduced. Acid is produced from d-ribose, d-glucose, d-fructose, d-maltose. l-arabinose, d-galactose, d-mannose, *N*-acetyl-glucosamine, aesculin, and gluconate are weakly fermented. Acid is not produced from glycerol, erythritol, d-arabinose, d-xylose, l-xylose, d-adonitol, methyl *β*-d-xylopyranoside, l-sorbose, l-rhamnose, dulcitol, inositol, d-mannitol, d-sorbitol, methyl-*α*-d-mannopyranoside, methyl *α*-d-glucopyranoside, amygdalin, arbutin, salicin, d-cellobiose, d-lactose, d-melibiose, d-sucrose, d-trehalose, inulin, d-melezitose, d-raffinose, starch, glycogen, xylitol, gentiobiose, d-turanose, d-lyxose, d-tagatose, d-fucose, l-fucose, d-arabitol, l-arabitol, 2-ketogluconate, and 5-ketogluconate. Dextran is not produced from sucrose. Growth occurs at 10–37 °C, but not 40 °C. Growth is observed at pH 4.0–8.0 and in the presence of 5% (w/v) NaCl. Cells do not contain *meso*-diaminopimelic acid in peptidoglycan.

The type strain, OKN36^T^ (= JCM 37107^T^ = DSM 118293^T^), was isolated from the bark of *Entada phaseoloides* collected at Amami Island, Japan, in 2020. The genome size of the type strain is 2.3 Mbp, and its DNA G + C content of the genome is 46.8%. The GenBank/EMBL/DDBJ accession numbers for the 16S rRNA gene and the genome sequences of strain OKN36^T^ are LC810165 and BQXN01000001-BQXN01000074, respectively.

## Supplementary Information

Below is the link to the electronic supplementary material.

Supplementary file1 (PDF 317 KB)

## Data Availability

The GenBank/EMBL/DDBJ accession numbers for the 16S rRNA gene and the genome sequences of strain OKN36^T^ are LC810165 and BQXN01000001-BQXN01000074, respectively.

## Abbreviations

ANI: Average nucleotide identity
dDDH: Digital DNA–DNA hybridization
MRS, de Man: Rogosa and Sharpe
