# Supplementary material for: Furfurilactobacillus entadae sp. nov., Isolated from Bark of Entada phaseoloides
Source: Curr Microbiol. 2025 Aug 29;82(10):480. doi: 10.1007/s00284-025-04450-4 (PMC12397155; doi:10.1007/s00284-025-04450-4)
Supplement: Supplementary file 1 — Supplementary file1 (PDF 317 KB) [file 284_2025_4450_MOESM1_ESM.pdf]

Supplemental Information

Furfurilactobacillus entadae sp. nov., isolated from bark of *Entada phaseoloides*

Shunya Suzuki [a,b], Karin Okano [a], Mizuna Tamaki [a], Yoshimasa Tsujii [a], Akihito Endo [c], and Akinobu Kajikawa [a]

[a] Department of Agricultural Chemistry, Graduate school of Tokyo University of Agriculture, 1-1-1 Sakuragaoka, Setagaya, Tokyo 156-8502, Japan.
[b] Bioproduction Research Institute, National Institute of Advanced Industrial Science and Technology, 1-1-1 Higashi, Tsukuba, Ibaraki 305-8566, Japan
[c] Department of Nutritional Science and Food Safety, Faculty of Applied Bioscience, Tokyo University of Agriculture, 1-1-1 Sakuragaoka, Setagaya, Tokyo 156-8502, Japan

***Corresponding author**
Akinobu Kajikawa, a3kajika@nodai.ac.jp

This file includes

- Supplementary Figure S1

Supplemental Figure

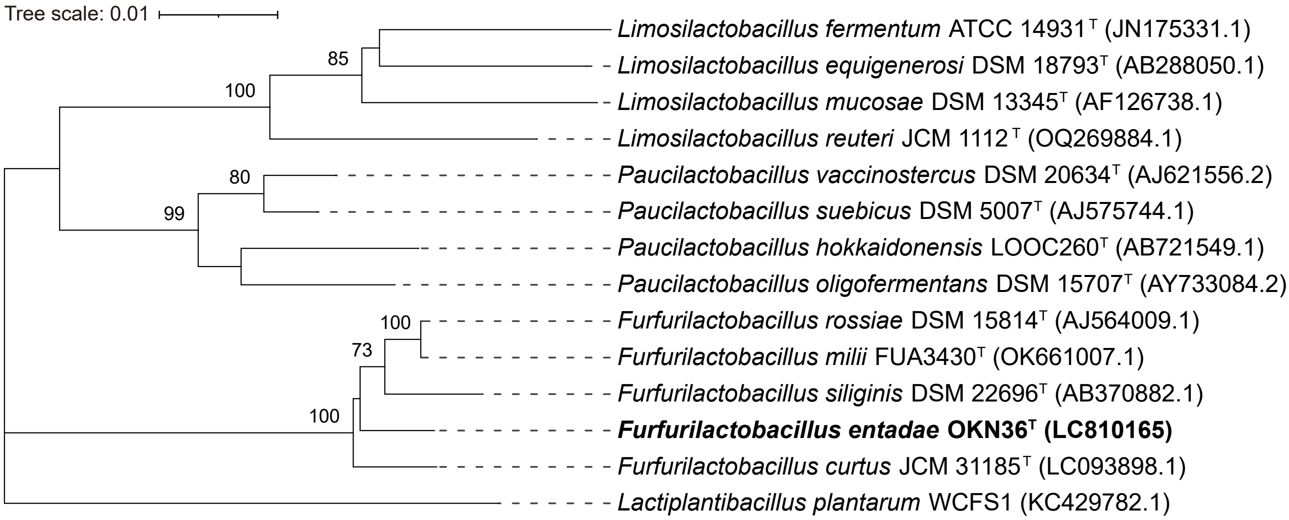

Fig. S1. Phylogenetic tree of *Furfurilactobacillus entadae* OKN36^{T} and related taxa based on 16S rRNA gene sequences. The tree was constructed by using the neighbour-joining method. The values on the branches are bootstrap support from 1000 rapid bootstrapping replicates, and only values over 70% are indicated. *Lactiplantibacillus plantarum* WCFS1 (KC429782.1) was used as an outgroup. The scale bar means substitution per site.
